# Cystoscopic removal of transvaginal mesh: Long‐term outcomes

**DOI:** 10.1002/bco2.254

**Published:** 2023-05-23

**Authors:** Katherine Anderson, Marie‐Aimée Perrouin‐Verbe, Lily Bridgeman‐Rutledge, Rachel Skews, Hashim Hashim

**Affiliations:** ^1^ Bristol Urological Institute, Southmead Hospital Bristol UK; ^2^ Bristol Medical School University of Bristol Bristol UK

**Keywords:** cystoscopic, erosion, mesh, outcomes, transvaginal

## Abstract

**Objectives:**

This study's aim is to evaluate the long‐term quality of life and functional outcomes following cystoscopic excision of stress urinary incontinence (SUI) and pelvic organ prolapse (POP) mesh extruded into the urinary tract in women.

**Patients and Methods:**

A retrospective chart review was performed of all cases of cystoscopic removal of extruded mesh at our high‐volume tertiary care centre between April 2013 and August 2021. Postoperative patient‐reported outcomes were collected via questionnaires: Urogenital Distress Inventory Short Form (UDI‐6), EQ‐5D‐5L Visual analogue scale, ICIQ‐Satisfaction (ICIQ‐S) and additional questions regarding postoperative sexual function.

**Results:**

During the study period, 27 women with a median age of 61 years (45–87) underwent cystoscopic mesh removal surgery using either Ho‐YAG laser (56%) or bipolar loop resection (44%). The most common presentation of mesh extrusion was recurrent urinary tract infections (67%). Other presenting complaints were pain (41%), urinary urgency ± incontinence (41%) and voiding difficulties (18%). Long‐term follow‐up outcomes from 20 patients (median follow‐up: 24 months) showed that mesh removal was rated successful by 80%, and 100% would choose to have the surgery again if in the same situation. Recurrent SUI was reported by 45% of respondents, and urinary urge incontinence was found in 50%. For patients who answered the sexual function questions, 50% reported improved sexual function postmesh removal (6/12).

**Conclusions:**

Cystoscopic removal of extruded female SUI and POP mesh is associated with high patient satisfaction and low morbidity in appropriately selected patients at 2‐year median follow‐up. A patient‐centred shared decision‐making process is essential in counselling patients regarding options and expected outcomes following mesh removal surgery.

## 1 INTRODUCTION

Transvaginal synthetic mesh has been commonly used to treat stress urinary incontinence (SUI) and pelvic organ prolapse (POP). The main types include retropubic midurethral mesh tension‐free vaginal tapes (TVT), transobturator mesh tapes/slings (TOT), single‐incision mini‐slings, and transvaginal POP mesh kits.^1^ Mesh extrusion into the urinary tract may occur in 5% of cases and can lead to serious problems of lower urinary tract symptoms (LUTS), haematuria, pain, recurrent urinary tract infections (UTIs), difficulties voiding, urinary incontinence and dysuria^2^ Patients may also experience a change in sensation in the leg, vagina, perineum or elsewhere.^3^ Tijdink et al. found that pain was the most common complaint of mesh erosion. These symptoms can significantly reduce quality of life (QoL) and can occur in approximately 10% of women who undergo mesh surgery.^4^ Some countries have recently recommended ‘high vigilance’ or restricted use of transvaginal POP and/or SUI mesh.^5^

Patients with complications may be offered surgical mesh removal via cystoscopic, laparoscopic, robotic‐assisted and/or an open approach.^6^ Open surgery includes partial or complete removal of mesh components.^7^ Tijdink et al. found that there was no difference in symptom relief between patients who underwent open partial versus open complete excision of mesh, in the short term. However, for those who had had POP mesh, recurrence of POP was more common after complete excision.^4^ This group also found that complications seemed to be more frequent in those who had complete mesh excision, although this difference was not statistically significant. There is certainly potential for significant morbidity from open surgery that should not be underrated, and it may not be feasible in all patients, but equally leaving mesh behind may cause problems in the future and make full mesh removal difficult.

Cystoscopic removal is done by intravesical resection of the mesh with electrode loop and transurethral resection/excision or ablation using a holmium laser.^8^ The advantages of endoscopic removal are that it may carry a lower morbidity risk, with shorter operating times, less blood loss and faster recovery time.^9^, ^10^ However, patients are more likely to require further intervention or repeat procedures, and it may not improve certain symptoms such as pelvic pain. Endoscopic mesh removal may have a relatively good success rate in the short term as was shown in a recent systematic review by Sobota et al., wherein only 22% (collated sample size = 41) of patients required a repeat cystoscopic treatment of their eroded mesh.^11^ However, the median follow‐up time was 6 months ± 17.0 (range 1–65 months), highlighting that long‐term evidence was limited.

The objectives of this work were to assess the postoperative course and complications of patients who underwent cystoscopic excision of urinary tract extruded mesh as well as the patient‐reported QoL and functional outcomes at long‐term follow‐up. We aim to utilise this information to improve patient counselling regarding management options for patients who have urinary tract complications from transvaginal SUI and/or POP mesh and to add to the growing body of evidence in the literature regarding best management practices for mesh complications.

## 2 PATIENTS AND METHODS

This research was registered as a quality improvement audit at our institution. The study group consisted of all women who underwent cystoscopic mesh removal surgery using laser or transurethral resection at our high‐volume tertiary care referral centre from April 2013 to August 2021. Retrospective chart review was undertaken to collect demographic, perioperative and follow‐up details. Primary outcomes were time to recurrence of mesh erosion and patient‐reported quality of life outcome measures (PROMs) at time of telephone survey. Specific PROMs collected were via a composite questionnaire including validated questionnaires:

1. Urogenital Distress Inventory Short Form (UDI‐6): Six specific questions regarding LUTS. Total score is transformed to score 0–100 with higher scores indicating worse distress.
2. EQ‐5D‐5L Visual analogue scale: Patient perception of their current health status on a scale of 0 (worst) to 100 (best).
3. ICIQ‐Satisfaction (ICIQ‐S): Satisfaction with surgical outcomes (0–24) and overall satisfaction with surgery (0–10), higher scores indicate higher satisfaction.
4. Additional questions regarding postoperative sexual function.

Descriptive statistics were performed using IBM SPSS Statistics, Version 27.0.

### 2.1 PRE‐OPERATIVE PLANNING AND OPERATIVE TECHNIQUE

Initial patient consultation took place in the Urology department at our centre. The patient's symptoms and investigations were reviewed, and surgical options were outlined. Cases were discussed in a multidisciplinary team (MDT) meeting. After discussion, the outcome was communicated to the patient and preferred course of management confirmed. A shared informed decision‐making process was used to aid the patient in deciding on the extent of mesh removal and surgical approach employed. This included offering full or partial removal of the mesh and the risks associated with each procedure.

All cystoscopic mesh removal was performed by one functional urology subspecialist or their senior trainee with appropriate supervision. Preoperative antibiotics were administered, and all cases were completed under general or spinal anaesthetic.

For bladder mesh erosion, either endoscopic bipolar loop resection or laser ablation was utilised. Bipolar loop resection was using a 26Fr sheath and continuous irrigation. Laser ablation was using a Ho‐YAG laser (fibre size: 550 or 1000 μm) through a laser working element The eroded mesh plus any calcifications were destroyed with the laser. The site of extrusion was also ablated deeper than the visible mesh to encourage scar formation to heal over the previous extrusion site.

Any urethral mesh erosion was exclusively managed via laser ablation rather than bipolar loop in a similar fashion making sure that no mesh was visible in the intraluminal urethral wall.

All patients had clinical follow‐up to assess amelioration of their symptoms including a flexible cystoscopy at 3–6 months from their procedure to assess for recurrence.

## 3 RESULTS

During the 8‐year study period, 70 women presented with mesh erosion into the urinary tract that elected for surgical excision. Of those, 27 women with a median age of 61 years (range 45–87 years) selected transurethral mesh removal surgery for mesh extrusion into the urinary tract (Table 1).

**TABLE 1:** Patient demographic information.

	*n* = 27
Median age in years (range)	61 years (45–87)
ASA grade (mode, range)	2 (1–4)
Median BMI (range)	27.6 (23–40)
Median time between mesh insertion to removal surgery	9 years (1–17)
*Co‐morbidities:*	
Fibromyalgia or chronic pain syndrome	22%
Irritable bowel syndrome	7%
Anxiety and/or depression	33%
Diabetes	7%

Presenting complaints leading to recognition of urinary tract mesh extrusion included recurrent urinary tract infections (*n* = 18, 67%), pain (*n* = 11, 41%), urinary urgency or urgency incontinence (*n* = 11, 41%) and voiding difficulties (*n* = 5, 18%).

Operative technique applied was either Ho‐YAG laser destruction of mesh (*n* = 19, 56%) or bipolar loop resection/fulguration (*n* = 15, 44%). The most common subtype of mesh encountered was retropubic TVT for SUI (Table 2). Extrusion into the bladder was most common.

**TABLE 2:** Mesh excision: Intra‐operative information.

Mesh type (*n* = 33)^a^	
Prolapse mesh	9% (3)
Transobturator mesh tape (TOT)	33% (11)
Retropubic tension‐free vaginal tape (TVT)	55% (18)
Unknown	3% (1)

Erosion location	
Bladder	44% (15)
Urethra	32% (11)
Both bladder and urethra	21% (7)
Unknown	3% (1)

^a^ Six patients had more than 1 type of transvaginal mesh implanted.

Length of stay in hospital following cystoscopic mesh removal was ≤1 day (IQR 0–4) in 87% (27/31) of cases. Thirty‐day complication rate was 10% (all were Clavien‐Dindo 1). Postoperatively, 26% (8/31) of patients did not require a catheter for bladder drainage, 26% had a catheter for 1–7 days and 48% had a temporary urethral catheter for more than 1 week.

On follow‐up cystoscopy, 26% (7/27) of patients had recurrence of mesh extrusion after first cystoscopic treatment and chose to undergo another endoscopic mesh removal procedure. The rate of progression from endoscopic treatment to requiring a more invasive open partial or total mesh removal surgery was 15% (4/27); all of these were SUI meshes.

Long‐term QoL and functional outcomes were obtained via telephone surveys with response rate of 74% (20/27) after a median follow‐up time of 24 months (IQR 7–74 months).

Total transformed UDI‐6 score was deemed symptomatic if greater than 33.3.^12^ Sixty‐five percent of survey respondents were asymptomatic at time of follow‐up (*n* = 13/20). Recurrent SUI occurred in 45% of respondents, and urgency urinary incontinence was reported by 50% (scored ≥2 on respective UDI‐6 questions). Among patients who had pre‐operative pelvic or bladder pain, they attributed to the mesh, 6/8 (75%) reported pain improvement (≤1 on pain‐specific UDI‐6 question). There were two patients without a recorded history of pre‐operative pain who reported current moderate bother from lower abdominal, pelvic or genital pain postmesh removal (score 2 on pain‐specific UDI‐6 question). One of these two patients had undergone open total mesh removal surgery following the endoscopic mesh removal.

**FIGURE 1:**
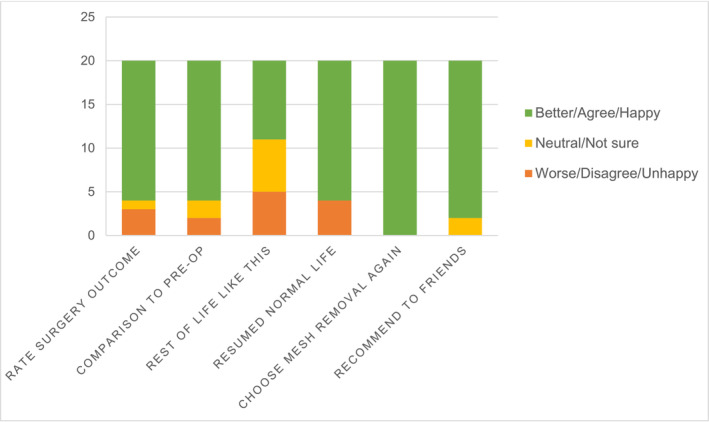
ICIQ‐Satisfaction (ICIQ‐S) outcomes questions survey results (*n* = 20).

**FIGURE 2:**
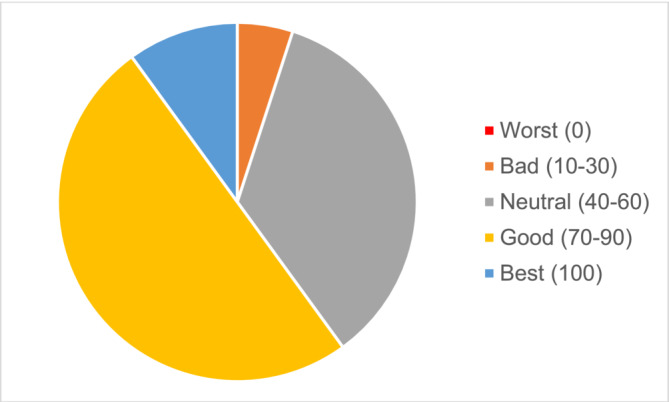
EQ‐ED‐5L Visual analogue scale (VAS) score results (*n* = 20).

ICIQ‐S Outcome section survey results found that mesh removal was rated successful by 80% of respondents, 90% would recommend the surgery to others in the same situation and 100% would have the surgery again if in the same situation (Figure 1). Overall satisfaction with cystoscopic mesh removal surgery was high with 80% of respondents rating ≥7/10 satisfaction. Patients self‐rated their current health status on the EQ‐ED‐5L visual analogue scale of at least 70 on a scale of 0–100 in 60% of the respondents (Figure 2).

Of the 20 survey responders, 12 answered at least one of the two questions specifically targeted at sexual function after mesh removal (response rate = 60%). The first question asked to patients was to compare their sexual life prepartial and postpartial mesh removal surgery; the second asked for frequency of pain during sexual intercourse, reduced sexual desire and/or difficulties reaching climax. Fifty percent of respondents reported improved sexual function at long term follow‐up compared with premesh removal (6/12), 33% reported no significant change in sexual life and 17% had worsened overall sexual life since surgery. Moderate to severe bother from sexual dysfunction such as pain, reduced desire or difficulties reaching climax was reported by 30% of respondents to the question (3/10).

## 4 DISCUSSION

There is a growing body of evidence that complications following transvaginal SUI and POP mesh can be delayed in presentation and have a significant impact on QoL.^13^ Mesh extrusion into the urinary tract can be particularly problematic as patients may suffer from recurrent UTIs, haematuria, worsening LUTS and pain before complications are recognised. Although removal/resection of the mesh via the least morbid means possible may seem like an ideal solution, the long‐term outcomes are not well‐elucidated in the literature with validated patient‐reported outcome measures. A recent systematic review found endoscopic management to have high success rates from 92% to 98% for laser, transurethral resection or laparoscopic scissor use, with low associated morbidity.^8^ Patient number per individual case series was small ranging from 3 to 23 patients each. Success was defined as no further erosion seen on last follow‐up, but long‐term PROMs were not evaluated. Recent work by Allagany, Dekalo, and Welk reported upon the largest single series of endoscopic laser ablation of SUI mesh extruded into the urethra.^14^ For the 29 women in their series, they used the UDI‐6 to evaluate PROMs with a median follow‐up length of 3.7 years. They concluded that there was minimal associated morbidity when the Ho‐YAG laser was used for extruded urethral mesh, with long‐term acceptable UDI‐6 scores. Our study also utilised the UDI‐6 as well as the ICIQ‐S (Outcome score), the EQ‐ED‐5L VAS, and questions particularly focused on sexual function. Sexual dysfunction can be overlooked in patients who have had complications following transvaginal surgery when the focus is on urinary tract symptoms.

In our series, the median time from mesh insertion surgery to recognition and management of mesh extrusion was 9 years which is slightly longer than in some previously reported work.^14^, ^15^ A prolonged lead time to extrusion may create issues for expedient diagnosis as the original surgeon who inserted the mesh may have moved practices or retired in that time and records could be difficult to acquire. Both patient and health care provider awareness of the potential complications from transvaginal mesh are critical to reduce delays in diagnosis. Patient groups have reported feeling unheard and dismissed so it is important to note that mesh extrusion into the urinary tract may present with a variety of symptoms such as LUTS or pain.^16^ In our patients, the most common presenting complaint leading to discovery of mesh extrusion was recurrent UTI.

Although sexual dysfunction is a known possible complication from mesh insertion, there is limited evidence that this problem improves after mesh removal.^17^ Only 60% of our survey responders answered the questions on sexual function; however, this could be because they are no longer sexually active so that may have steered them to answer, ‘not applicable’. Of the responders, 50% of the women reported improved sexual function currently compared with before mesh removal. Further work focused on sexual function after mesh excision surgery is needed.

Overall, cystoscopic mesh removal was rated as highly satisfactory, as exemplified by 100% of responders answering that they would have mesh excision again if in the same situation (ICIQ‐S, Figure 1). There were no complications greater than Clavien‐Dindo 1 reported in the 30‐day postoperative period.

This study does have several limitations. The surgeries were all completed at a tertiary care centre by urologists who have extensive experience in dealing with mesh complications. The applicability therefore of these findings to centres with less experienced providers may be cast in doubt. However, as endoscopic procedures using the laser or resectoscope are ubiquitous in most urologists' practices, the same principles are applicable. This makes this type of mesh removal in contrast to invasive open or laparoscopic routes more accessible across jurisdictions. The overall low complication rate also favours this approach as a technique that urologists outside of tertiary centres may find doable as long as patients are offered all surgical options and a shared decision‐making process is followed. Secondly, historically, it was not routine to administer PROMs to patients prior to mesh removal surgery so we were unable to draw direct comparisons premesh and postmesh excision. As well, specific surgical approach utilised (laser or resectoscope) was left to the discretion of the surgeon at the time of the case so the data are somewhat heterogeneous. This could be viewed as a strength of the work as it lends itself to real‐life applicability wherein urologists must be adept at using various types of endoscopic tools as called for by the case encountered.

As there are currently no validated mesh complication PROMs, we utilised a composite questionnaire for LUTS and patient surgery outcome satisfaction. Future research should strive to utilise mesh complication‐specific questionnaires once validated.

In the absence of high‐level evidence, a shared‐decision process between the surgical team and the patient is key to mesh removal surgery. Patients need to be presented with all surgical options and the pros and cons of each including the lack of long‐term data. In some countries, there are now mesh complication centres that can offer patients all forms of treatment in a multidisciplinary approach.^3^

In conclusion, we reported on a large series of cystoscopic mesh removal cases for women with mesh extrusion into the urinary tract including long‐term follow‐up PROMs. Transurethral surgery for SUI and POP mesh extrusion was associated with high patient satisfaction, low morbidity and high success rates. Half of the patients had improved sexual function following removal of the mesh. A patient‐centred shared decision‐making process is essential in counselling patients regarding options and expected outcomes following mesh removal surgery.

## AUTHOR CONTRIBUTIONS

**Katherine Anderson:** data collection, data analysis and manuscript preparation. **Marie‐Aimée Perrouin‐Verbe:** data collection and manuscript revisions. **Lily Bridgeman‐Rutledge:** data collection, data analysis and manuscript preparation. **Rachel Skews:** data collection and manuscript revisions. **Hashim Hashim:** data collection and manuscript revisions.

## CONFLICT OF INTEREST STATEMENT

Katherine Anderson, Marie‐Aimée Perrouin‐Verbe, Lily Bridgeman‐Rutledge, Rachel Skews and Hashim Hashim have no relevant conflicts of interest to declare.
